# A Feedback Model for Applied Research on Tobacco Control

**Published:** 2006-03-15

**Authors:** Gail G Sneden, Amy S Gottlieb-Nudd, Nell H Gottlieb, Philip P Huang

**Affiliations:** Public Health Promotion and Program Development, Department of Kinesiology and Health Education, University of Texas at Austin; Public Health Promotion and Program Development, Department of Kinesiology and Health Education, University of Texas at Austin, Austin, Tex; Public Health Promotion and Program Development, Department of Kinesiology and Health Education, University of Texas at Austin, Austin, Tex; Texas Department of State Health Services, Chronic Disease Prevention Branch, Austin, Tex

## Abstract

Although the health communication program feedback cycle is frequently referenced, the steps for moving between or within the sections of the model in a public health environment are rarely described. We detail the process by which the Texas Tobacco Research Consortium implemented the stage of "assessing effectiveness and making refinement" and expanded it to include a program assessment feedback model.

Tools were developed to move the consortium through five stages of the expanded program assessment feedback model: 1) formulate research questions using logic models to identify key evaluation items, 2) format data displays from multiple data sources to address research questions, 3) use a facilitated group process to present and review research findings, 4) prepare group recommendations, and 5) involve local partners to translate recommendations into practice.

The process allowed us to sift through a large volume of information and prepare data-based program recommendations. A Web-based reporting system provided timely access to community-based program activity data and process indicators that, when linked to logic models, provided actionable items for program improvement. Partnerships among researchers and state and local practitioners created the conditions for implementing the recommendations.

Program changes included revisions to program materials, target audiences, and evaluation instruments for a community-based tobacco-cessation campaign. The systematic approach allowed translation of research into practice and should be applicable to other areas of population-based health promotion.

## Introduction

Public health promotion programs should be dynamic. They should change based on the findings of previous program activities and emerging research. To effect these changes, public health leaders should collaborate with academic partners to link evaluation findings with local program revisions through a continuous health communication program process, or feedback cycle (1), which is the backbone of a public health knowledge-management system. The health communication program feedback cycle developed by the U.S. Department of Health and Human Services includes four stages: 1) planning and strategy development; 2) developing and pretesting concepts, messages, and materials; 3) implementing the program; and 4) assessing effectiveness and making refinements (1).

The feedback cycle has been frequently referenced (2,3) and used as the basis for implementing numerous health communication programs, but the steps for moving between or within the sections in a public health environment are rarely described. Likewise, methods for accessing, organizing, and interpreting a large volume of information in a timely and effective manner — and among highly diverse groups of stakeholders — are rarely discussed. Theories and models of health promotion that integrate systems can help close the gap between research and practice, and they need to be shared by all parties seeking to interpret data (4-6). According to Best et al, the particular model used may be less important than the process of working with an explicit structure toward the development of a learning culture (5).

The Texas Tobacco Research Consortium was established in 2000 with funding from the Texas Tobacco Settlement, a settlement between Texas and the tobacco industry in 1998 (7). University researchers, originally from six academic institutions along with researchers and practitioners from the state public health department and the American Cancer Society worked together to gather baseline data, assist with program implementation, and evaluate a state-mandated tobacco pilot program. The partners met regularly throughout 2005 to design and conduct tobacco program evaluation and surveillance surveys, with each university carrying out different aspects of the pilot project under its contract with the public health department.

## Expanded Program Assessment Feedback Model

In this article, we expand on the fourth step of the feedback cycle: assessing effectiveness and making refinements. The five stages of the expanded program assessment feedback model are as follows: 1) formulate research questions using logic models to identify key evaluation items, 2) format data displays from multiple data sources to address research questions, 3) use a facilitated group process to present and review research findings, 4) prepare group recommendations, and 5) involve local partners to translate recommendations into practice.

We describe the supporting processes, including use of logic models and group facilitation, in selecting, formatting, and interpreting data to improve state and local programs. We use the Texas tobacco-cessation program implemented by the Texas Tobacco Research Consortium to illustrate how data collected from state agencies and multiple academic partners can be used to improve program performance. The expanded program assessment feedback model that evolved is available from the University of Texas Web site (8).

The expanded feedback model relies on data collected by researchers at the state, national, and international levels. State data are archived with the state health department, synthesized, and submitted in a concise format for review by members of a multidisciplinary team. Recommendations for program change reference research, including the *Guide to Community Preventive Services* (9) and the Centers for Disease Control and Prevention's (CDC's) *Best Practices for Comprehensive Tobacco Control Programs* (10). Final recommendations are transferred through multiple channels (e.g., program materials, training, newsletters, contracts) to promote effective implementation.

The expanded feedback model was developed in 2001, but the consortium was unable to implement it successfully right away. The vision made sense conceptually, but the initial implementation raised questions. Which data among various surveys, instruments, and other items should be used? Given the diverse sampling procedures, which data applied to a given community? How could outcomes be linked to program inputs? How could diverse data sources be brought together in a timely manner that made sense to representatives from multiple academic disciplines and cultural backgrounds? A knowledge-management system (11) was needed to address these issues. The knowledge-management system used in this project included people (i.e., state agencies and independent researchers from five Texas universities and the American Cancer Society); processes (i.e., use of logic models, facilitated group process, formatted data displays, and meetings); and technology (i.e., a Web-based program management and tracking system). The knowledge-management system allowed the consortium to actualize the expanded feedback model, and it is described on the University of Texas Web site (8).

### Program management and tracking system

Web-based reporting systems help standardize local data collection and build consensus for strategic goals and objectives. The advent of the Web-based Program Management and Tracking System (PMATS) (8) in March 2003 provided timely access to community-based tobacco-cessation program activity data and was an important link for large-scale feedback models. A user-friendly format allowed contractors and grantees to enter an activity report within minutes. PMATS reflected the strategic plan for tobacco control in Texas and target goals outlined in CDC's *Best Practices*. It recorded information on activity type, activity location, the number of people affected, demographic information on people affected, and a narrative description of the activity.

The capacity to generate community-based program activity reports was necessary to create an effective feedback cycle. Research indicates that frequency and encouragement of feedback are positively associated with computer-based monitoring practices (12). The reporting component of PMATS provided instant feedback on local data.

### Using logic models to create a common vision

We initially developed logic models for each program goal for the purpose of designing survey instruments (8). The logic models later proved to be effective in communicating program activities and guiding data analysis. The logic models also allowed us to maximize use of approximately 25 evaluation and surveillance instruments with more than 1000 items collected by independent Texas researchers working on tobacco prevention and control through such diverse fields as juvenile justice, health psychology, human development, health communication, and public health promotion.

## Application of Program Assessment Feedback Model to Tobacco Control

The first four steps in the program assessment feedback model occurred during face-to-face meetings of consortium members, who included researchers and health department professionals. Typically held every other month, the 4-hour meetings were meticulously planned with process and outcome agendas (8). Each consortium member typically received an outcome agenda before the meeting with instructions on selecting and organizing the data they had collected (8). The advance agenda allowed consortium members to process different types of research along with key findings related to the logic models.

### Step 1: formulate research questions

The tobacco-cessation logic model developed by the consortium (8) includes a series of key indicators, including public exposure to mass media campaigns, the level and type of media buys, and the number of referrals and calls to the Quitline, a free telephone counseling service established in Texas by the American Cancer Society in 2000 for people who wanted to stop smoking. Before each meeting of the consortium, meeting planners reviewed the indicators and developed an agenda that detailed the research questions. Although other indicators were reviewed, we focus on two research questions in this article: 1) how effective were mass media campaigns in generating calls to the Quitline? and 2) to what extent did community-based tobacco-cessation activities increase calls to the Quitline?

### Step 2: format data displays

Several weeks before the consortium meeting that focused on these two questions, researchers were asked for one- to three-page data displays that addressed the questions. One data display showed the relationship between the launch dates of mass media campaigns and county and state Quitline call volume as reported by the American Cancer Society (8). Another showed the relationship between mass media campaigns (billboards, flyers, and community presentations urging smokers to call Quitline) and the number of community-based tobacco-cessation activities in a pilot county that had high rates of lung cancer and stroke according to PMATS (8). Both data displays are available on the Web site of one of the academic partners at the University of Texas (8).

The two data displays cover five mass media campaigns that ran in Texas during 2002 through 2004: the Great American Smoke-Out (November 2002 and November 2003), Quit and Win (April 2003 and April 2004), and Yes You Can! (January 2004). The Great American Smoke-Out is an annual event sponsored by the American Cancer Society (13). Quit and Win is a mass media campaign that urges smokers to give up tobacco products in exchange for a chance to win a prize (14). Yes You Can! is a mass media campaign developed by the Texas Department of State Health Services that urges the target audience of blue-collar workers to stop smoking (15).

### Step 3: use a facilitated group process

A skilled facilitator, knowledgeable about the data, helped guide consortium meetings. Based on timed agendas, members presented their findings. After considering multiple data sources and linking results to the cessation logic model, consortium members generated recommendations on ways to sustain and improve tobacco-cessation programs*.*

When they examined the data on Quitline call volume, number of tobacco-cessation activities, and run dates of the various mass media campaigns, for example, consortium members noted early spikes in call volume during October and November 2002 when three campaigns advertising the Quitline and the need to stop smoking were aired simultaneously (8). Although the Texas Quitline had been operating for more than 2 years, a campaign advertising the service was conducted by the American Cancer Society in fall 2002. During fall 2002, the Texas Department of State Health Services purchased air time to broadcast Quitting Takes Practice, a mass media campaign produced by the California Department of Health Services (16). Also, the early spikes in 2002 took place during the Great American Smoke-Out. The same spikes did not occur during the Great American Smoke-Out in 2003. The Yes You Can! campaign appears to have generated neither an increase in Quitline calls nor community tobacco-cessation activities when conducted independently of the other activities.

Consortium members also noted that the highest level of community-based tobacco-cessation program activity in the pilot county occurred in April 2004. During spring 2004, a mass media campaign and community-based programs in the pilot county focused on an antismoking campaign to counter tobacco industry advertisements urging people to smoke and included efforts to publicize free nicotine replacement therapy (NRT), the Quitline number, and the opportunity to win a prize for giving up smoking through a local Quit and Win contest.

The second highest level of community-based tobacco-cessation activity in the pilot county occurred in November 2003, during the Great American Smoke-Out. This, however, did not coincide with an increase in call volume. In April and May 2004, the pilot county experienced a spike in call volume that was independent of state call volume and paralleled an increase in local program activity. Findings from a follow-up survey of smokers who entered the Quit and Win contest revealed that more than 90% of the smokers who registered for the contest did not call the Quitline, and fewer than 25% were aware of the free NRT (17). The survey was conducted 2 months after the contest ended.

### Step 4: prepare group recommendations

During the first years of the consortium (2000 through 2002), researchers included tobacco-cessation program recommendations in their end-of-year reports to the state health department. The recommendations, a few of which were included in the report to the state legislature, were all too often embedded in lengthy reports, written in terminology appropriate for research publications, and effectively lost to local communities. Review, discussion, and development of recommendations during group meetings proved to be important at state and local levels. The process not only created increased awareness of the findings but also assisted with the implementation of program changes and promoted the cross-referencing of local findings with national guidelines (9).

About twenty recommendations were generated and grouped into three categories: change mass media messages or delivery channels, expand community awareness, and modify evaluation. Recommendations included actions for program developers, community partners, and evaluators at multiple levels and are detailed on the Web site (8).

### Step 5: involve local partners

Our approach to working with community-based partners has grown out of experience. Participatory processes are often cited as the solution — and a necessity — for the challenge of engaging local community support (18). Participants are guided to identify strategies for moving recommendations into practice through their agency action plan as well as general recommendations for program change. The latter includes integration of recommendations into contractor performance objectives and trainings; revision of mass media messages, delivery channels, and program resources; and linking the timing of surveys to key program activity dates. However, the majority of our local partners demonstrated greater interest in program delivery rather than evaluation and a desire to use their limited time toward improved program implementation. They wanted to know whether their actions had made a difference and how their efforts had contributed to addressing concerns of the state legislature.

Community partners reviewed the recommendations and provided more suggestions for what was needed to increase calls to the Quitline. At one meeting, we discussed new ways to deliver program messages, identified better ways to work together, and initiated study groups to carry out projects in the pilot county. The first study group selected research on smoke-free restaurants as a way to reduce both smoker and nonsmoker exposure to tobacco smoke. A participatory process created a consultation calendar in which local partners identified topics they believed would be most beneficial in their communities. Online newsletters provided additional information and resources to the communities (8).

Community leaders and contractors now meet to review selected data and recommendations from content experts. Examples of key questions posed in local group consultations include the following: What needs to stay the same? What needs to change? How do we use this information to create change? The framework is used to guide the discussion and is supplemented by worksheets designed to help analyze program messages, target audience, and delivery channels and formulate recommendations for improving the frequency and intensity of messages**.**

## Lessons Learned

The process used to expand the fourth stage of the health communication program feedback cycle evolved during a 5-year period. The process may work for others who manage a dynamic and diverse body of public health research with local implications. Following are key lessons learned during the process:

- Create a common understanding of which data are being collected and why. Program logic models are tools that allow researchers and practitioners to formulate common research questions and identify the most relevant data. The wealth of data and cross-tabulations allow for a wide range of research questions. Focus on one or two key research questions within the group, such as: How effective were program activities in achieving the intended outcomes? What did we learn from the campaign? How can we strengthen implementation strategies to increase tobacco cessation using the evidence available?
- Develop a Web-based program activity reporting system, such as PMATS, for maintaining timely access to process data that can be used to identify actions for program improvement. A Web-based program, along with a central database and report archives, creates organizational memory and a data-retention system.
- Format selected data related to the research question so that key stakeholders can easily understand it. Typically, a wealth of information is embedded within various databases — the limited amount extracted for local program planning should not interfere with professional publications and basic research reports.
- Interpret findings together. Use facilitated face-to-face meetings to review the data. Plan meetings well in advance, and alert all parties to the kind of information that will be needed as well as the format desired for delivering the information. Develop recommendations as a group.
- Invest in the implementation and communication of recommendations at all levels. Work collaboratively to identify how the recommendations relate to program content, implementation, and evaluation. Communicate progress on the recommendations.
- Create a knowledge-management system that includes knowledge creation, retention, transfer, and use.

The expanded program assessment feedback model is another tool for public health promotion and is not limited to tobacco-cessation programs. Effective use of the model depends on the timely availability of locally relevant data and a knowledge-management, retention, and transfer process. When linked to a knowledge-creation system, such as a public health research consortium, and an organization dedicated to improving performance by leveraging current and future knowledge, data-based program development can become a reality.
